# A novel desmocollin-2 mutation reveals insights into the molecular link between desmosomes and gap junctions

**DOI:** 10.1016/j.hrthm.2011.01.010

**Published:** 2011-05

**Authors:** Katja Gehmlich, Pier D. Lambiase, Angeliki Asimaki, Edward J. Ciaccio, Elisabeth Ehler, Petros Syrris, Jeffrey E. Saffitz, William J. McKenna

**Affiliations:** ⁎ Institute of Cardiovascular Science, University College London, and The Heart Hospital, London, United Kingdom; † Department of Pathology, Beth Israel Deaconess Medical Centre, Harvard Medical School, Boston, Massachusetts; ‡ Department of Medicine, Division of Cardiology, Columbia University Medical Center, New York, New York; § King's College London, Randall Division of Cell and Molecular Biophysics and Cardiovascular Division, BHF Centre of Research Excellence, London, United Kingdom

**Keywords:** Cardiomyopathy, Conduction, Connexin43, Desmocollin-2, Desmoglein-2, Desmosome, Functional studies, Gap junction, Mutation, Plakoglobin, ARVC, arrhythmogenic right ventricular cardiomyopathy, Cx43, connexin43, DAPI, 4′,6-diamidino-2-phenylindole, DSC2, desmocollin-2, DSG2, desmoglein-2, DSP, desmoplakin, GFP, green fluorescent protein, GST, glutathione-S-transferase, ICS, intracellular cadherin segment, PG, plakoglobin, PKP2, plakophilin-2, RV, right ventricle, YFP, yellow fluorescent protein

## Abstract

**Background:**

Cellular adhesion mediated by cardiac desmosomes is a prerequisite for proper electric propagation mediated by gap junctions in the myocardium. However, the molecular principles underlying this interdependence are not fully understood.

**Objective:**

The purpose of this study was to determine potential causes of right ventricular conduction abnormalities in a patient with borderline diagnosis of arrhythmogenic right ventricular cardiomyopathy.

**Methods:**

To assess molecular changes, the patient's myocardial tissue was analyzed for altered desmosomal and gap junction (connexin43) protein levels and localization. *In vitro* functional studies were performed to characterize the consequences of the desmosomal mutations.

**Results:**

Loss of plakoglobin signal was evident at the cell junctions despite expression of the protein at control levels. Although the distribution of connexin43 was not altered, total protein levels were reduced and changes in phosphorylation were observed. The truncation mutant in desmocollin-2a is deficient in binding plakoglobin. Moreover, the ability of desmocollin-2a to directly interact with connexin43 was abolished by the mutation. No pathogenic potential of the desmoglein-2 missense change was identified.

**Conclusion:**

The observed abnormalities in gap junction protein expression and phosphorylation, which precede an overt cardiac phenotype, likely are responsible for slow myocardial conduction in this patient. At the molecular level, altered binding properties of the desmocollin-2a mutant may contribute to the changes in connexin43. In particular, the newly identified interaction between the desmocollin-2a isoform and connexin43 provides novel insights into the molecular link between desmosomes and gap junctions.

## Introduction

Desmosomes provide a rigid link between neighboring cells, which allows them to withstand mechanical strain. In these structures, the extracellular portions of desmosomal cadherins mediate cell adhesion across the cell borders, while their intracellular modules bind to the plaque proteins plakoglobin (PG) and plakophilin, which in turn bind desmoplakin (DSP).^1,2^ The latter is associated with intermediate filaments, which establish a mechanical continuum across cells.^3^ The importance of this mechanical integrity is underlined by human disease manifestations. Mutations in desmosomal genes can result in cardiac disease, including arrhythmogenic right ventricular cardiomyopathy (ARVC), cutaneous disorders (palmoplantar keratoderma), and multitissue syndromes (e.g., Naxos disease).^4^

In cardiac desmosomes, two desmosomal cadherins are expressed: desmocollin-2 (DSC2) and desmoglein-2 (DSG2).^5^ Both have overlapping functions in binding PG and plakophilin-2 (PKP2), believed to be mediated by a conserved region in their cytoplasmic portion, the intracellular cadherin segment (ICS).^1^ Desmocollins are characterized by two splice variants (a form and b form) with different cytoplasmic portions.^6^ The DSC2a protein contains the full ICS domain, whereas this module is significantly truncated in the DSC2b isoform. The ratio of isoforms appears to be controlled in a tissue-specific manner,^7^ suggesting an important regulatory role for tissue integrity.

Desmosomal adhesion is a prerequisite for proper gap junction formation and maintenance at cell–cell contact sites.^8^ Connexin43 (Cx43) is the major ventricular gap junction protein, which ensures electrical coupling of neighboring cardiomyocytes.^9^ In cardiac disease, changes of Cx43 localization are often observed.^10–13^ This phenomenon has been demonstrated in ARVC patients bearing *PKP2* mutations.^14,15^ The pathologic process of gap junction remodeling is thought to contribute to a high risk for arrhythmias characterizing the condition.^16^ However, the molecular mechanisms of the impaired link between desmosomal function and gap junction remodeling are not fully understood.

We present the case of a patient with slow right ventricular (RV) conduction in the presence of a truncation mutation in *DSC2* and a missense variant in *DSG2*. Analysis of endomyocardial tissue from this patient revealed that molecular changes in protein expression and phosphorylation may precede an overt ARVC phenotype. Moreover, our functional studies on the mutant DSC2 protein shed light on how the DSC2a isoform may contribute to desmosome and gap junction interdependence by providing a direct binding interface for Cx43.

## Material and methods

### Clinical evaluation

The study was performed in accordance with the 1964 Declaration of Helsinki, and the study protocol was approved by the local ethics committee. All individuals provided informed consent. Clinical evaluation included history, physical examination, 12-lead ECG, signal-averaged ECG, two-dimensional echocardiography, and 24-hour ECG monitoring. The diagnosis of ARVC was based on the recent revision of the Task Force criteria.^17^

### Electrophysiologic study

The patient underwent noncontact mapping study^18^ of the RV to investigate endocardial activation and the morphology of noncontact unipolar electrograms in the triangle of dysplasia. Endocardial isochronal and isopotential maps were recorded in sinus rhythm and during steady-state pacing at the RV apex with a cycle length of 400 ms. Total RV endocardial activation times and maximum activation gradient were measured as previously described.^19,20^

### Histologic and immunohistochemical analysis of endomyocardial biopsy material

Snap-frozen RV biopsy samples were obtained and analyzed for protein expression by Western blotting. Formalin-fixed, paraffin-embedded material was stained with hematoxylin and eosin for histologic examination. Unstained slide-mounted sections were immunostained for N-cadherin (Sigma, UK), PG (Sigma, UK), and Cx43 (Zymed via Invitrogen, UK).

### Functional studies

Localization studies in HL-1 cells and neonatal rat cardiomyocytes as well as biochemical binding experiments were performed to assess the consequences of *DSG2* and *DSC2* mutations. Full experimental details are available in the Online Supplementary Material.

## Results

### Identification of a DSC2 mutation in a patient with RV conduction abnormalities

**Figure 1:**
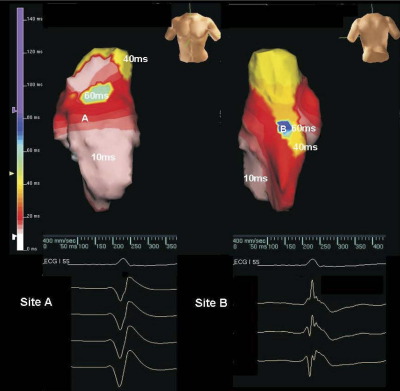
Right ventricular (RV) conduction abnormalities in the index patient. Isochronal maps of sinus rhythm are shown. The maps are orientated in the anterior **(A)** and posterior **(B)** views, respectively. The color scale represents timing of activation from onset of the QRS complex (red = early, purple = late). The total RV endocardial activation time was 82 ms, with latest site of activation in the posterior RV (site B). The electrograms in this area are fractionated compared to those in the anterior wall at site A. Similar fractionated electrograms were also identified in the posterolateral RV wall.

A 52-year-old woman presented with palpitations and a family history of sudden cardiac death (Figure S1). Her resting ECG showed sinus rhythm, PR interval of 190 ms, QRS duration of 84 ms, and prolongation of the terminal S wave (>55 ms) in lead V_1_ (Figure S2A). The signal-averaged ECG was negative for late potentials. Noncontact mapping of the RV demonstrated moderately prolonged total RV endocardial activation time of 82 ms in sinus rhythm versus <65 ms recorded in healthy controls.^20^ Furthermore, the maximum endocardial activation gradient was only 0.22 mm/ms, which is 40% of that recorded in healthy controls by our group using this technique (0.51 ± 0.15 mm/ms).^21^ Fractionated and low-amplitude electrograms also were identified in the posterior RV wall and posterolateral RV outflow tract segments (Figure 1).

**Figure 2:**
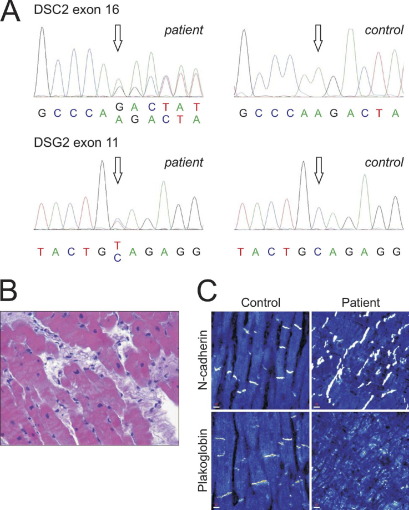
Histologic and immunohistological changes in the presence of desmosomal mutations. **A:** Sequence electropherogram of the heterozygous deletion in exon 16 of *DSC2* (c. 2553 delA, **top**) and of a heterozygous nucleotide change in exon 11 of *DSG2* (c. 1550 C>T, **bottom**) in the index patient (*open arrows,***left**) compared to a control individual (**right**). **B:** Interstitial and replacement fibrosis in a myocardial sample of the patient (original magnification ×40). **C:** Confocal immunofluorescence microscopy analysis of nonfailing control tissue and a myocardial sample from the index patient. The plakoglobin signal is strongly reduced at the intercalated disks of the index patient, whereas the adherens junction protein N-cadherin can be abundantly detected at the intercalated disks of both samples (see also Figure S3). Scale bar at *lower left* in each panel represents 10 μm.

Although the patient did not fulfill the diagnostic criteria for ARVC (Table S1), clear evidence of slow conduction and fragmented electrograms was seen in the patient's RV, and this feature is compatible with a subclinical ARVC phenotype.^22^ These sites of electrogram fractionation (Figure 1B) indicated that local conduction heterogeneities were present that could promote conduction block and reentry leading to ventricular arrhythmia. ARVC mutation screening was performed, and two mutations were identified:

1 A heterozygous deletion was found in exon 16 of *DSC2* (c. 2553 delA). This frameshift mutation is predicted to introduce a premature stop codon at position 855, truncating the last 47 amino acids of the *DSC2*a isoform (*DSC2* Q851fsX855, Figure 2A).
2 A heterozygous nucleotide change was identified in exon 11 of *DSG2* (c. 1550 C>T), resulting in an alanine to valine change at amino acid position 517 (*DSG2* A517V). None of the changes was found in 450 controls.

The 18-year-old daughter of the index patient was also positive for both sequence changes (Figure S1). Clinical evaluation showed T-wave inversion in leads V_1_ and V_2_ on the ECG (Figure S2B). However, the patient had no other diagnostic features suggestive of ARVC. Like her mother, she had a borderline diagnosis of ARVC (Table S1).

Family evaluation revealed that the sudden cardiac death in the family was unrelated to desmosomal mutations (Figure S1).

### Changes in myocardium in the presence of desmosomal mutations

Endomyocardial biopsy of the patient showed evidence of interstitial and replacement fibrosis (Figure 2B). Furthermore, the immunoreactive signal for PG was severely depressed at the cardiac intercalated disks, whereas the adherens junction protein N-cadherin was clearly present (Figure 2C). These observations are in agreement with molecular changes occurring in the myocardium of ARVC patients.^23^

**Figure 3:**
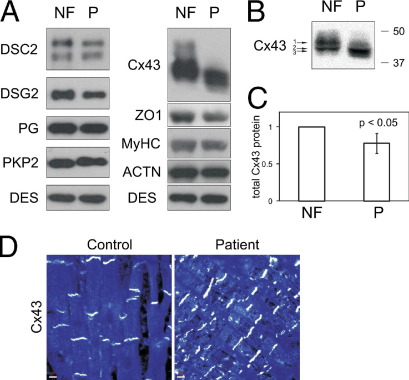
Molecular changes in desmosomal and gap junction proteins in the patient's myocardium. **A:** Western blot analysis in the patient (P) compared to nonfailing control tissue (NF). Samples were assayed for desmosomal proteins and connexin43 (Cx43). Blotting for desmin (DES), sarcomeric markers alpha-actinin (ACTN), and myosin heavy chain (MyHC) served as loading controls. **B:** High-resolution Western blotting of Cx43 identified two different bands in the control sample (*arrows 1* and *2*), whereas the dominating band in the patient sample migrated even faster (*arrow 3*) The position of marker proteins (molecular weight in kilodaltons) is indicated. **C:** Reduced total Cx43 protein levels in the patient sample (Cx43 normalized to desmin, NF set to 1, N = 3). **D:** Confocal immunofluorescence microscopy analysis for Cx43 in control and patient myocardial sample showed normal localization of Cx43 at the intercalated disks. Scale bar at *lower left* in each panel represents 10 μm. DSC2 = desmocollin-2; DSG2 = desmoglein-2; DSP = desmoplakin; PG = plakoglobin; PKP2 = plakophilin-2.

Western blotting for desmosomal components showed minimal reduction of both DSC2 and DSG2 expression levels (Figure 3A). Because the truncated DSC2a protein has electrophoretic mobility similar to that of the DSC2b form, no quantitative information about isoform ratios (DSC2a/DSC2b) could be made. Other desmosomal proteins (including PG) and desmin were at control levels. The total protein content of the gap junction protein Cx43 was mildly reduced in the patient's sample (by approximately 20%, Figure 3C), whereas the band appeared to be migrating differently (Figure 3A). High-resolution analysis identified two different Cx43 bands for the control sample and one major band for Cx43 in the patient sample (Figure 3B). This band had an even slightly higher electrophoretic mobility than the lower band of the control sample, which is indicative of differences in Cx43 phosphorylation.^24^ The gap junction–associated protein ZO-1 was also found to be slightly reduced.

Immunofluorescence analysis of Cx43 in the endomyocardial biopsy sample showed a normal localization pattern (Figure 3D). Collectively, analysis of the patient's myocardial tissue suggested that the desmosomal mutations may reduce Cx43 protein levels and phosphorylation but not overall distribution.

### Localization studies of DSC2 and DSG2 mutations

**Figure 4:**
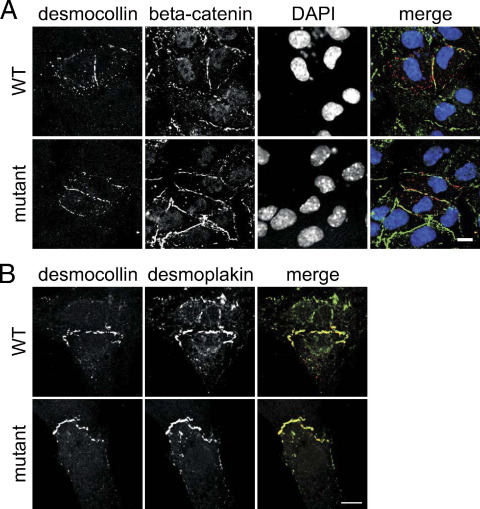
Desmocollin-2 (DSC2) Q851fsX855 mutant is incorporated into cardiac desmosomes. **A:** Localization of DSC2a wild-type (WT) and mutant proteins in transiently transfected HL-1 cells **(first row),** counterstained with the cell–cell contact marker beta-catenin **(second row),** nuclei visualized with 4′,6-diamidino-2-phenylindole (DAPI; **third row**), merged images (**fourth row:** DSC2 *red,* beta-catenin *green,* DAPI *blue*). Scale bars represent 10 μm. Both WT and mutant DSC2 were primarily found at the cell–cell contacts. **B:** Localization of DSC2a WT and mutant proteins at the intercalated disks of transfected neonatal rat cardiomyocyte **(first row),** counterstained with DSP **(second row),** merged images (**third row:** DSC2 *red,* DSP *green*). Scale bar represents 10 μm. The DSC2a mutant protein was incorporated normally into cardiac desmosomes.

Normal localization of the mutant DSC2 and DSG2 proteins was observed at the desmosomes of the cardiac cell line HL-1 (Figures 4A and S4C). Moreover, the mutant proteins were targeted to the intercalated disks of neonatal rat cardiomyocytes, where they co-localized with the desmosomal proteins (Figures 4B and S4D). Hence, any pathogenic action of the mutant proteins would arise from altered protein function (e.g., binding properties) at the cardiac desmosomes. Altered binding properties for the DSG2 A517V change were not identified (Figure S4).

### Functional consequences of the truncated cytoplasmic domain in DSC2a

**Figure 5:**
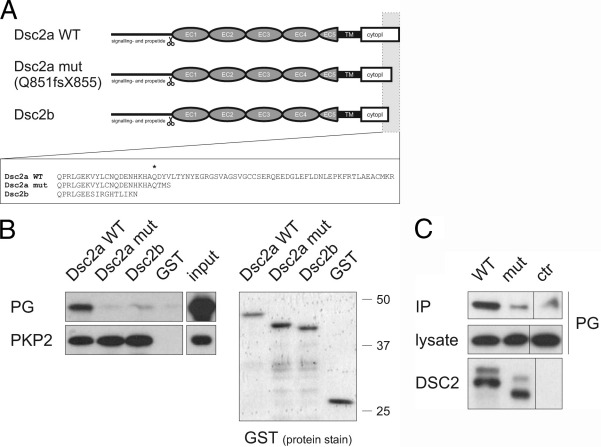
DSC2a Q851fsX855 mutant is impaired in binding desmosomal plakoglobin (PG). **A:** Consequences of the DSC2 Q851fsX855 mutation for cytoplasmic region (cytopl) of the DSC2a isoform. DSC2a (wild-type [WT] and mutant) as well as DSC2b are shown schematically. The amino acid sequence of the intracellular cadherin segment for each DSC2 protein is given. *Asterisk* marks Q851. Adapted from Awad et al.^5^ EC = extracellular cadherin domain; TM = transmembrane domain. **B:** Binding of PG and plakophilin-2 (PKP2) to the cytoplasmic region of DSC2a WT, DSC2a mutant, and DSC2b protein in GST pulldown assays, together with input control. Glutathione-*S*-transferase (GST) protein inputs are also shown (marker protein positions on the *right*). Whereas all DSC2 forms bound PKP2, only DSC2a WT bound also to plakoglobin. **C:** Impaired binding of DSC2a mutant full-length protein to plakoglobin in co-immunoprecipitation experiments. IP = immunoprecipitated proteins; ctr = control experiment (empty vector).

The mutation in exon 16 of *DSC2* is predicted to have no direct consequences for the DSC2b splice variant (Figure 5A). In contrast, a large portion of the conserved ICS in the DSC2a isoform is predicted to be truncated by the mutation. Because the ICS is thought to mediate binding to other desmosomal components, binding of the DSC2a mutant to the desmosomal plaque proteins PG and PKP2 was tested. In GST pulldown experiments, the cytoplasmic portion of DSC2a wild-type (WT) bound to both PG and PKP2 from rat heart lysate, whereas DSC2b bound only to PKP2 (Figure 5B). No binding of the mutant DSC2a protein to PG was observed, and binding to PKP2 was unaffected. Moreover, the mutant DSC2a did not bind to a DSP construct (Figure S5A). Using full-length DSC2a proteins in co-immunoprecipitation experiments, the mutant form showed strongly reduced binding to PG compared with its WT counterpart (Figure 5C). These experiments suggest that Q851fsX855 may interfere with intradesmosomal protein interactions. In agreement with the intracellular position of the truncation, binding to DSG2 (mediated by the extracellular portion) was preserved (Figure S5B).

**Figure 6:**
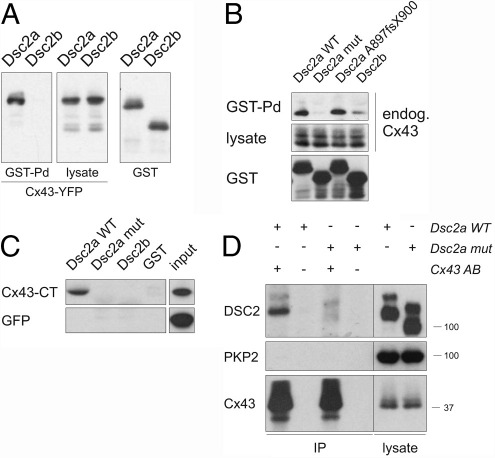
Novel interaction between DSC2a and connexin43 (Cx43), which is abolished in the mutant DSC2a Q851fsX855 form. **A:** COS-1 cells coexpressing glutathione-*S*-transferase (GST) fusion proteins for the cytoplasmic region of DSC2a (wild-type [WT]) and DSC2b, together with Cx43 yellow fluorescent protein (YFP) fusion protein were subjected to GST pulldown assays. Cx43 bound to DSC2a but not to DSC2b. **B:** DSC2a WT, Q851fsX855 mutant, A897fsX900 variant, and DSC2b cytoplasmic region were fused to GST and expressed in COS-1 cells. Binding to endogenous Cx43 was observed with DSC2a WT but not for DSC2b. The mutation in DSC2a abolished binding to Cx43, whereas the A897fsX900 variant was functionally equivalent to WT. Lysate levels indicate that DSC2a predominantly binds the hypophosphorylated form of Cx43. **C:** Binding of Cx43 cytoplasmic tail (CT) to DSC2a WT, DSC2a mutant, and DSC2b protein in GST pulldown assays. Cx43 cytoplasmic tail was expressed as a green fluorescent protein (GFP) fusion protein in COS-1 cells and assayed for binding to bacterially expressed DSC2 cytoplasmic region GST fusion proteins (see Figure 5B). Binding was only observed for DSC2a WT and not for the DSC2a mutant or DSC2b. There was no binding to GFP alone **(second row). D:** DSC2a mutant protein fails to bind Cx43 in co-immunoprecipitation experiments. Transfected full-length DSC2a WT and mutant were assayed for binding to endogenous Cx43, which was immunoprecipitated (where indicated, + in AB). Samples omitting the antibody served as negative controls. Endogenous plakophilin-2 (PKP2) was not detectable in immune complexes. The position of the marker bands is indicated on the right. IP = immunoprecipitated proteins.

The observed changes in Cx43 total protein level and phosphorylation in the patient's myocardium (Figure 3) suggested a link between Q851fsX855 and the gap junction protein. Accordingly, we examined the possibility of a direct interaction between DSC2a and Cx43. Indeed, the cytoplasmic portion of WT DSC2a was found to co-sediment with Cx43-YFP in GST pulldown assays, but no binding was observed between DSC2b and Cx43-YFP (Figure 6A). Moreover, DSC2a WT bound to endogenous Cx43, and again the mutant DSC2a protein and DSC2b did not bind (Figure 6B).

The binding site within Cx43 was mapped to its cytoplasmic tail (Figure 6C), and only DSC2a WT bound to it, whereas no binding of either the mutated DSC2a protein or the DSC2b isoform was detected. Using full-length DSC2a WT and mutant protein in co-immunoprecipitation experiments, binding to endogenous Cx43 was confirmed for DSC2a WT, whereas no interaction was detectable between the mutant DSC2a and Cx43 (Figure 6D).

These results not only point toward a novel direct interaction between DSC2a and Cx43 but also indicate that the abolished interaction between the mutant DSC2a protein and Cx43 could contribute to the gap junction protein changes observed in the patient's myocardium.

## Discussion

Beyond mechanical adhesion, desmosomes have been implicated in signaling processes such as apoptosis, cell migration, and proliferation.^1,2^ In particular, a functional link between desmosomes and gap junctions has become evident through cardiac disease. In ARVC, mutations in genes coding for cardiac desmosomal proteins affect gap junction morphology^10^ and function,^14,15^ and this pathologic situation is thought to contribute to the ventricular arrhythmias that characterize the disease. In Naxos disease, gap junction remodeling has been shown to possibly precede fibrofatty replacement of cardiomyocytes.^11^

In this study, a patient who presented with delayed RV endocardial conduction and local fractionation of electrograms in the triangle of dysplasia was found to be a carrier of two desmosomal mutations. Despite the patient's benign phenotype, pathologic changes were evident at the molecular level. Immunoreactive signal for PG was remarkably reduced at the intercalated disks (Figure 2). This feature was found to characterize remodeling processes occurring in the myocardium of ARVC patients independent of the underlying gene mutation.^23^ The observation that total PG levels were unchanged (Figure 3A) supports the hypothesis that PG is redistributed to other cellular pools.^25^

Moreover, changes in the main cardiac gap junction protein Cx43 were evident in the patient. Reduced Cx43 protein levels were detected (Figure 3A), and, more importantly, a shift in electrophoretic mobility was observed (Figure 3B). In the patient's sample, faster migrating Cx43 was more prominent, suggesting a lower proportion of the highly phosphorylated protein.^26^ The biologic relevance of this posttranslational modification at multiple sites in Cx43 is complex, as phosphorylation events are thought to regulate gap junction assembly, disassembly, and channel activity.^24^ The slight reduction in gap junction protein ZO-1 indicates that the entire structure might be affected.

A series of *in vitro* experiments performed provides insight into the molecular mechanisms that could underlie the observed changes. Functional studies on the *DSG2* A517V change failed to identify any pathogenic potential (Figure S4). However, we cannot exclude completely that this conservative change contributes to the phenotype. The truncated DSC2a Q851fsX855 protein was also found to be normally incorporated into cardiac desmosomes (Figure 4); however, it completely abolished binding to PG and DSP, whereas binding to PKP2, which occurred in both DSC2 isoforms, was not affected (Figures 5 and S5). It is likely that the presence of the mutant DSC2a protein in the cardiac desmosomes reduces the capacity of these structures to retain PG, which could contribute to the observed redistribution of PG from junctional pools (Figure 2), as shown for other ARVC causing DSC2 mutations.^27^ In particular, nuclear translocation of nonjunctional PG may aberrantly modulate Wnt signaling pathways, driving adipogenesis and fibrogenesis.^25,28^ Due to the limited availability of patient material, we could not stain for other desmosomal proteins in our myocardial samples. Therefore, the assumption that PKP2 still is present at the intercalated disks is based solely on *in vitro* binding data (Figure 5) and is somewhat speculative.

The truncation mutation in *DSC2* only affects the DSC2a isoform, whereas the DSC2b splice variant is not affected. A recent report suggested low expression levels of the DSC2a isoform in the heart and indicated that truncation of the last five amino acids of DSC2a by the A897fsX900 variant is not sufficient to cause ARVC^29^ despite reduced binding to PG.^27^ In contrast, in our patient with slow conduction and borderline diagnosis of ARVC, truncation of a substantial portion of the DSC2a ICS domain by the Q851fsX855 mutation causes complete loss of binding to PG and DSP. Moreover, our functional data shed new light on the unique cellular functions of the DSC2a splice isoform in cardiac tissue. Only this isoform (in its WT form), and not its DSC2b counterpart, provides a direct physical link to Cx43. Whereas the interaction is not affected by the A897fsX900 variant, the Q851fsX855 mutation abolishes this cellular function of DSC2a (Figure 6B). This may, among many other factors,^30,31^ contribute to the conduction abnormalities observed in the patient.

Oxford et al^32^ reported an interaction between desmosomal PKP2 and Cx43. However, the binding profiles of WT DSC2a and DSC2b do not suggest that the DSC2a Cx43 interaction is mediated by PKP2 (Figure 5). Moreover, PKP2 was not detectable in our immunocomplexes using a Cx43 antibody (Figure 6D). This discrepancy may be attributable to differences in experimental setups. Additionally, the multimolecular complex analyzed by Oxford et al might also have contained DSC2a or other bridging molecules. Nevertheless, the observation that knock-down of PKP2 affects Cx43 expression and localization as well as cell coupling properties supports our hypothesis that desmosomal proteins are essential for proper gap junction function. Down-regulation of PKP2 affected localization of DSP^32^ and, most likely, other desmosomal proteins, such as DSC2, as well. Hence, the lack of other desmosomal components at the intercalated disks, rather than the loss of PKP2 itself, could have triggered the redistribution and down-regulation of Cx43.

Despite the observed molecular changes and evidence of significant reductions in both RV endocardial conduction time and activation gradient (a surrogate of conduction velocity), no ventricular arrhythmia was evident in the patient. A similar phenomenon of significantly high “conduction reserve” has been reported in animal models. Mice with a heterozygous deletion of Cx43 in the heart have no overt phenotype, and a reduction of Cx43 protein down to 10% of normal levels is required to induce spontaneous arrhythmias.^33^ These data indicate that reduced Cx43 expression contributes to an optimal milieu that facilitates arrhythmogenesis in ARVC. However, a specific physiologic trigger (e.g., increased adrenergic tone) promoting ventricular ectopy or further modulation of the substrate by inflammatory processes may be required to increase the probability of a clinically important arrhythmic event. These modulations in the substrate are expected to increase the heterogeneities in conduction and repolarization kinetics in the ventricle promoting conduction block, reentry, and the generation of ventricular tachycardia and fibrillation.

Analysis of this patient with a subclinical ARVC phenotype provides valuable insight into disease mechanisms. Changes in gap junction protein Cx43 occur before any clinical manifestation significant enough to fulfill Task Force criteria for diagnosis but is, nevertheless, detectable utilizing high-density electrophysiologic mapping of the substrate. Moreover, the newly identified interaction between DSC2a and Cx43 may contribute to the interdependence of desmosomal integrity and gap junction function and thereby opens avenues for new therapeutic approaches in ARVC.
