# Supplementary material for: A novel desmocollin-2 mutation reveals insights into the molecular link between desmosomes and gap junctions
Source: Heart Rhythm. 2011 May;8(5):711–8. doi: 10.1016/j.hrthm.2011.01.010 (PMC3085091; doi:10.1016/j.hrthm.2011.01.010)
Supplement: Supplementary Material [file mmc1.pdf]

A novel desmocollin-2 mutation reveals insights into the molecular link between desmosomes and gap junctions

Supplementary material

Katja Gehmlich, Pier D. Lambiase, Angeliki Asimaki, Edward J. Ciaccio, Elisabeth Ehler, Petros Syrris, Jeffrey E. Saffitz, William J. McKenna

Supplementary Methods

Genetic screening

Genomic DNA was extracted from whole blood using QIAamp DNA Blood mini kits (Qiagen). For screening of *DSP, JUP* (coding for PG), *PKP2, DSG2, DSC2* and *GJA1* (coding for Cx43), primer pairs for all exons were used from flanking intronic sequences[1]. Polymerase chain reaction (PCR) amplification was carried out using standard protocols (AmpliTaq Gold, Applied Biosystems), and fragments with high GC content were amplified with the GC RICH PCR system (Roche). All primer sequences and PCR conditions are available on request. PCR products were subjected to direct sequencing in both directions on an Applied Biosystems 3130 Genetic Analyzer using BigDye Terminator chemistry (v3.1) and analyzed by Seqscape v2.5 software (Applied Biosystems). 450 unrelated healthy, ethnically matched volunteers served as controls.

Histological and Immuno-histochemical analysis of endomyocardial biopsy material

Ventricular biopsy samples from the RV septum obtained from the index patient during a right heart catheterization were initially snap frozen and later fixed in 10% non-buffered

formalin, embedded in paraffin, cut in 5 μm sections and stained with hematoxylin and eosin for histological examination. Unstained slide-mounted sections of paraffin-embedded tissue were immunostained and myocardial specimens obtained at autopsy from three age-matched individuals with no clinical history or pathological evidence of heart disease were subjected to the same staining protocols and served as controls[2]. Briefly: slide-mounted sections were deparaffinised, placed in citrate buffer (10 mmol/l, pH 6.0), and heated in a microwave oven until boiling for 10 min to enhance specific immunostaining. After being cooled to room temperature, the tissue sections were simultaneously permeabilised and blocked by incubating them in PBS containing 0.1 % Triton X-100 and 3 % normal goat serum. The sections were then incubated with the primary mouse monoclonal antibodies (mAB), anti-PG, anti-N-cadherin (both Sigma) or anti-Cx43 (Zymed), overnight at 4 °C, brought to room temperature, washed three times in PBS, and incubated with indocarbocyanine-conjugated goat anti-mouse or anti-rabbit IgGs (Jackson Immunoresearch) for 2 h at 25 °C. Immunostained preparations were analyzed by laser-scanning confocal microscopy (Sarastro Model 2000, Molecular Dynamics) as previously described[2].

Western blotting

Protein analyses by Western blotting in comparison to a non-failing control sample was performed as described[3] using following mABs: anti-DSC2/3 (Zymed), anti-PG (clone 15/γ-Catenin BD Biosciences), anti-PG (clone 15F11, Sigma), anti-DSG2 (clone DG3.10, Progen), anti-DSG2 (clone 6D8, abcam), anti-desmin (DEB5, Millipore), anti-Myosin (clone A4.840, Santa Cruz) and anti-Cx43 (clone CX-1B1, Zymed) as well as the following pR antibodies: anti-DSC2 (Prestige Antibody Ab2, HPA012615, Sigma), anti-Cx43 (Abcam), anti-desmin (Sigma), anti-ZO-1 (Zymed), anti- sarcomeric alpha-actinin[4]. Even loading and transfer was

verified by PonceauS stain (Sigma). Each protein was probed for in duplicates, using two different antibodies where possible, and one representative blot is shown.

For high resolution Western blotting of different Cx43 species the chemiluminescent signal (SuperSignal West Pico Chemiluminescent Substrate, Thermo Scientific) was recorded with the Molecular Imager Chemidoc XRS system using QuantityOne software (both Biorad).

For quantification of Cx43 in the patient sample, blots for Cx43 and desmin (loading control) were run in triplicates. Densitometric analysis was performed (ImageJ software), and the ratio of Cx43 to desmin expressed relative to the control sample (control set to 1). Data are visualised as mean +/- standard deviation. Student's t-test was performed and $p < 0.05$ considered being significant.

Generation of cDNA constructs

The A517V change was introduced into a full length DSG2 GFP fusion construct[5] with the QuikChange mutagenesis kit (Stratagene) according to the manufacturer's instructions using the following primer pair: 5'-gtatgtgaatgttactgtagaggacctggatggac-3', 5'-gtccatccaggtcctctacagtaacattcacatac-3'. Generation of wild-type (WT) full length DSC2a pCDNA3 has been described elsewhere[6]. The Q851fsX855 mutation was introduced as above using following primers: 5'-gaaaatcacaagcatgcccagactatgtcctgacatataa-3' and 5'-tatatgtcaggacatagtctgggcatgcttgtgattttc-3'. The pCDNA3 constructs coded for the human DSC2 proteins without any additional amino acids; these proteins could be detected with DSC2 antibodies (which do not cross-react with endogenous mouse or rat DSC2 protein).

A full length Cx43-YFP construct was kindly provided by Dr John Gomes (University College London, UK). The generation of a GFP fusion construct of the N-terminal portion of DSP (DSP-NTP) has been described elsewhere[6].

Cell culture and transient transfections

HL-1 cells (a kind gift of Dr R. Breckenridge, Centre for Clinical Pharmacology, University College London, UK) were cultured in Claycomb medium (Sigma), supplemented with 10 % fetal bovine serum (Sigma), 2 mM L-glutamine (Gibco), 100 units/ml penicillin, 100 µg/ml streptomycin (Gibco) and 100 µM noradrenalin (Sigma) on. HL-1 cells were transfected with 4 micrograms of DNA and Lipofectamine 2000 (Invitrogen) in fibronectin-gelatin (Sigma) coated 35 mm dishes according to the manufacturer's instructions. Primary cultures of neonatal rat cardiomyocytes (NRC) were established using the Neonatal Cardiomyocyte Isolation System from Worthington (Lakewood, NJ, USA). The cells were plated and maintained as described previously[4] and transfected on day 1 after plating using 5 micrograms of DNA and JetPrime transfection reagent (Autogen Bioclear).

Indirect immunofluorescence and confocal microscopy

Transfected cells were fixed with 4 % paraformaldehyde in phosphate buffered saline (PBS) for 10 min at room temperature, and subsequently permeabilized with 0.2 % Triton X-100 in PBS for 5 min. Cells were blocked with 10 % normal goat serum (Sigma) in PBS prior to incubation with the primary antibodies (mAB anti-DSC2/3, mAB anti-PG, pR anti-alpha-actinin, pR anti-DSP, Serotec, pR anti-beta-catenin, Sigma), diluted in 1 % bovine serum albumin in PBS, for 1 h at room temperature. After washing with 0.1 % Tween-20 in PBS, cells were incubated with Goat-anti-Mouse-antibody or Goat-anti-Rabbit-Antibody conjugated to fluorescent dyes (Jackson ImmunoResearch, following combinations: anti-rabbit Cy2 with anti-mouse Cy3). DAPI (Sigma) was used to visualize nuclei. After being washed, the cells were mounted in Tris-buffered glycerol with n-propyl gallate (Sigma) as anti-fading agent. Specimens were analyzed and documented on a confocal microscope Leica SPE (Leica), using a 63x/1.30 oil immersion lens and solid phase lasers.

GST-pulldown assays

Human sequences corresponding to cytoplasmic regions of DSC2a WT (amino acids (aa) 716-901), DSC2a Q851fsX855 (aa 716-855) and DSC2b (716-847) were cloned into pGEX6P1 (GE Healthcare). Protein expression in the BL21 Codon Plus strain (Stratagene) and purification with glutathione-sepharose (GE Healthcare) have been described elsewhere[6].

Protein-loaded beads were incubated with pre-cleared adult rat heart lysate (350 µg total protein) or COS-1 cell lysate (transfected with human Cx43 cytoplasmic tail construct (aa 228-382) in pEGFP-C1, or with empty vector, Clontech,) in GST-pulldown buffer (0.5 % NP-40, 20 mM Tris/HCl pH 7.5, 120 mM sodium chloride, 1 mM dithiothreitol, 1 mM sodium ortho-vanadate and protease inhibitors) for 1h on ice. GST alone served as a negative control. After washing three times with GST-pulldown buffer, the bound proteins were eluted with two-fold SDS-sample buffer. GST-fusion proteins were analysed by SDS-PAGE and visualized with InstantBlue (Generon), binding of YFP- or GFP-fusion proteins (using mAB anti-GFP, Roche), PG and PKP2 (multi-epitope cocktail, Progen) was detected by Western blotting. For comparison, diluted lysate samples (1 % of total input) were run as lysate controls on the same gel.

The same GST fusion constructs (together with DSC2a A897fsX900, aa 716-900) were also cloned into mammalian expression vector pEBG as above for expression as GST-fusion proteins in COS-1 cells[6]. The cells were transfected with 7.5 micrograms of each pEBG constructs alone in 100 mm dishes (to assay binding to endogenous Cx43). Alternatively, the pEBG constructs were transfected together with the Cx43-YFP or DSP-NTP-GFP, using 5 microgams of DNA each. After 48 h, the transfected COS-1 cells were harvested, lysed in GST-pulldown buffer for 20 min on ice, and extracts clarified by centrifugation at 50,000 g at 4°C for 15 minutes. GST-pulldown assays were performed by

addition of glutathione-sepharose for 1 h on ice and washed, eluted and analyzed as above, using anti-Cx43, anti-GFP and goat polyclonal anti-GST (GE Healthcare) antibodies.

Co-immunoprecipitations

COS-1 cells, transfected with full length DSC2a WT or mutant, or empty parental pcDNA3 vector, were lysed in ice-cold IP-buffer (0.5 % NP-40, 1 % Triton X-100, 20 mM Tris/HCl pH 7.6, 2 mM EDTA, 138 mM sodium chloride, 5 % glycerol, 5 mM dithiothreitol, 1 mM sodium ortho-vanadate and protease inhibitors, Roche) and incubated on ice for 60 minutes. Subsequently, the extract was clarified by centrifugation at 50,000 g at 4°C for 15 minutes. Immunoprecipitations were performed using 1 µg anti-DSC2/3 or anti-Cx43 in the presence of 1 % bovine serum albumin. Immuno-complexes were purified with Protein G sepharose (Sigma), washed three times with IP-buffer and the bound proteins were eluted with two-fold SDS-sample buffer and detected by Western blotting. For comparison, diluted lysate samples (1 % of total input) were run as lysate controls on the same gel.

To assess DSG2-DSC2 interactions, COS-1 cells were transfected with construct combinations as indicated and lysed in IP-buffer supplemented with 60 mM n-octyl-beta-D-glucoside. GFP proteins were precipitated with 1.5 µg anti-GFP as above.

Supplementary Figures

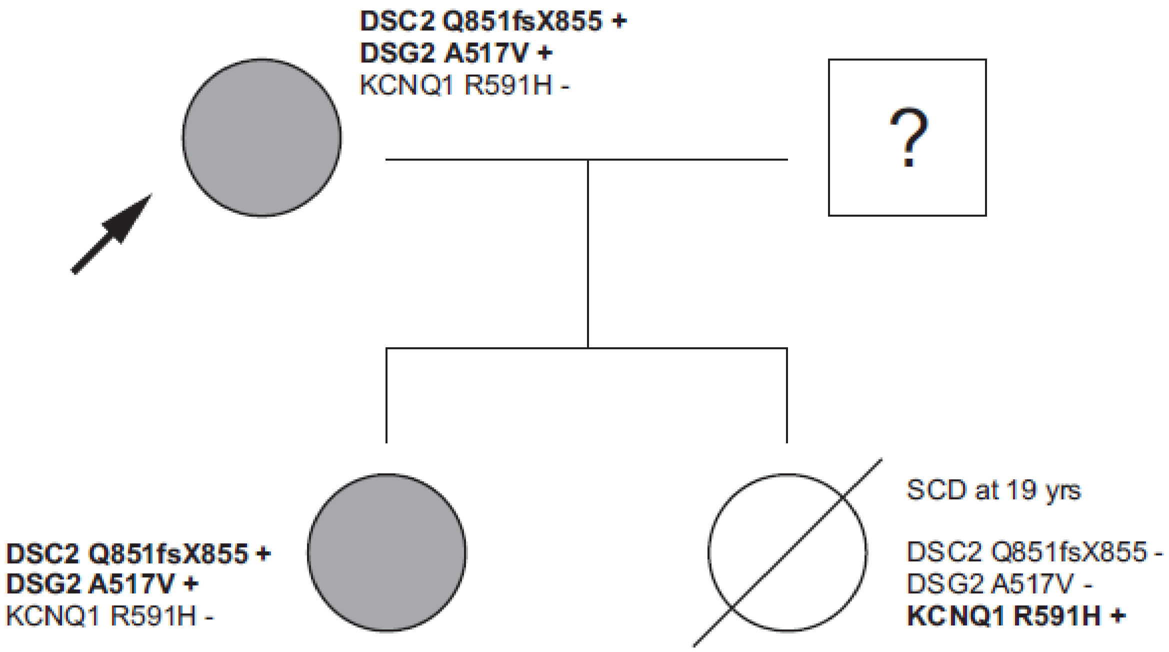

Figure S1:

Pedigree of the family: grey symbols indicate individuals with a borderline diagnosis of ARVC (see Table S1). Slanted bar indicates deceased individual (sudden cardiac death at the age of 19); squares indicate males; circles indicate females; plus (+) and minus (−) signs indicate the presence or absence of the listed mutations; arrow: index patient; question mark: the father was not available for clinical or genetic evaluation.

One daughter of the index patient had died suddenly at the age of 19 years. She was negative for both DSC2 and DSG2 changes. Instead, a heterozygous KCNQ1 R591H missense change was identified, suggestive for sudden cardiac death caused by long QT syndrome[7]. Neither the index patient nor her living daughter were found to carry this KCNQ1 mutation. Hence, the sudden cardiac death in the family was unrelated to the desmosomal mutations.

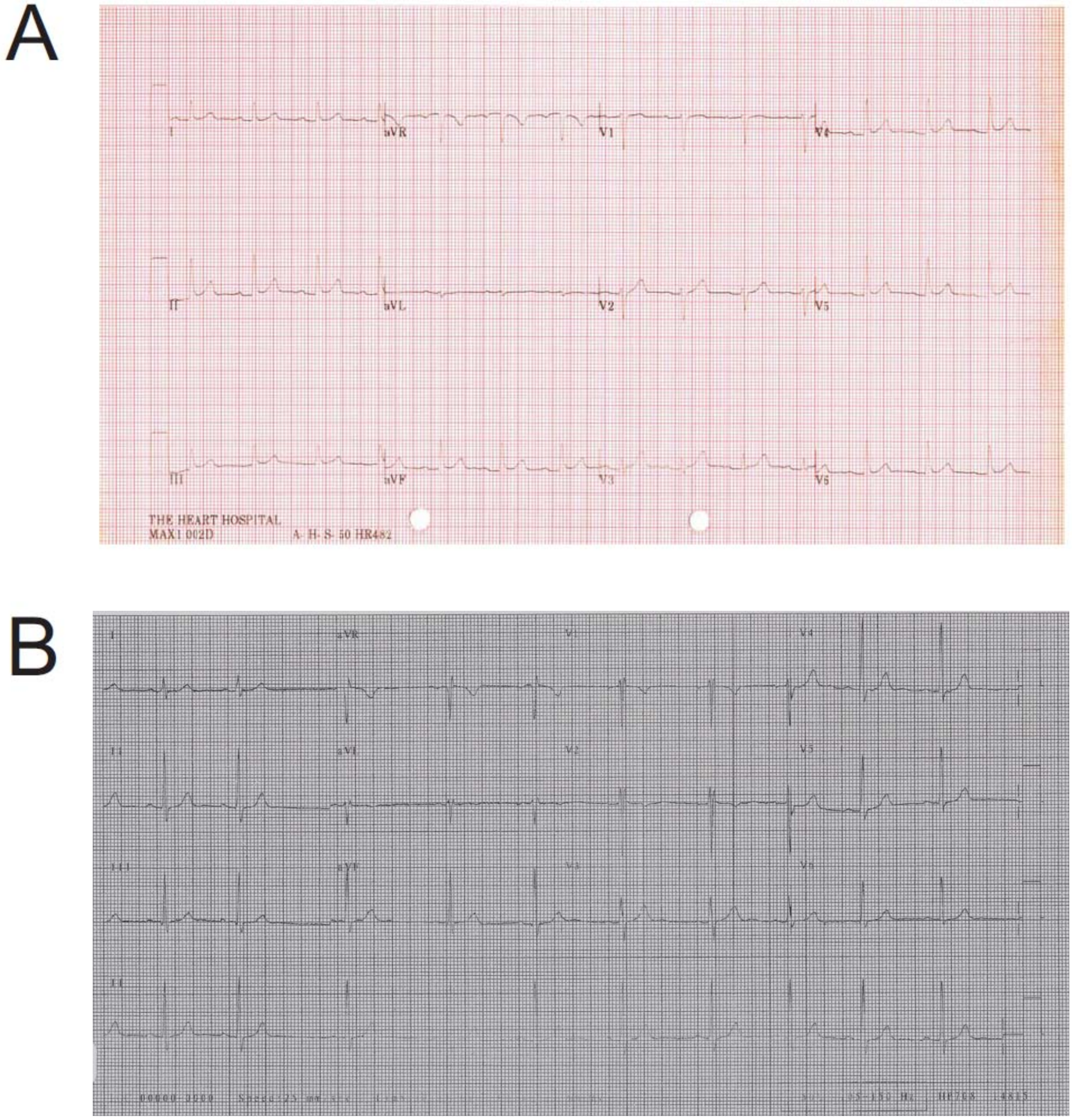

Figure S2:

A – Resting ECG of the index patient demonstrating prolongation of the terminal S wave (>55msec) in V1.

B – Resting ECG of the living daughter with T-wave inversion in V1 and V2.

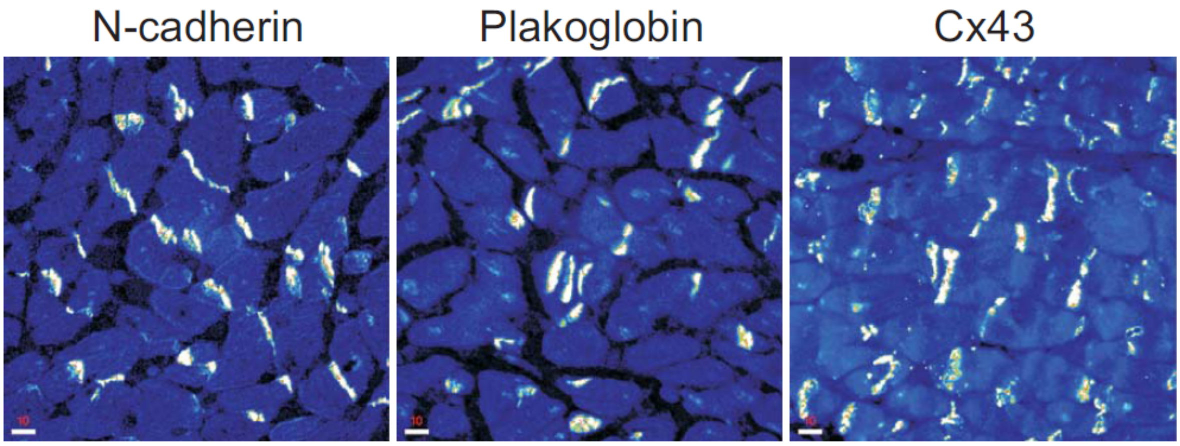

Figure S3:

Confocal immunofluorescence microscopy analysis of non-failing control tissue. For better comparison to the patient sample (see Figures 2 and 3), oblique sections of control tissue were selected. Intercalated discs are clearly identifiable. Scale bar at lower left in each panel represents 10 µm.

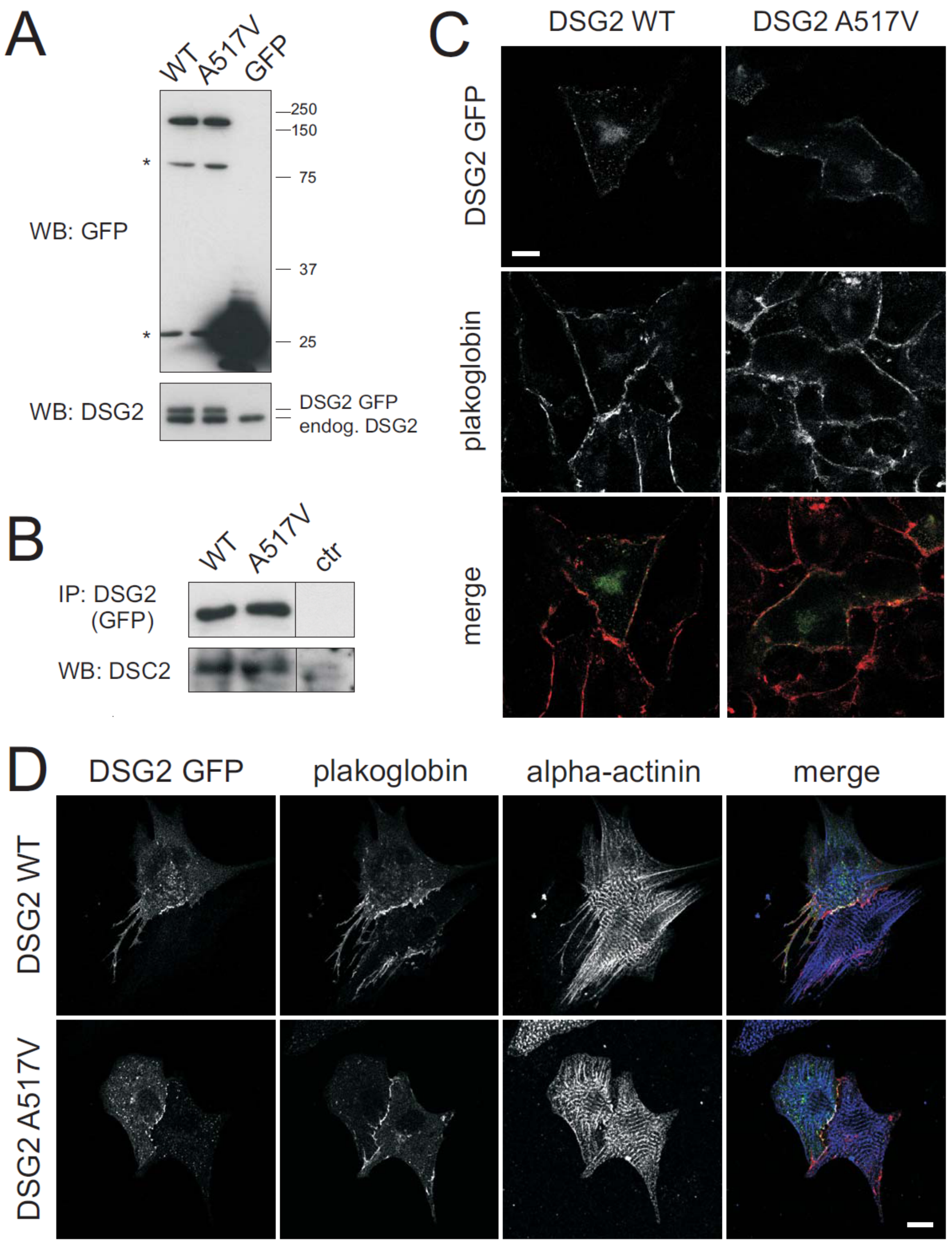

Figure S4: Functional studies on DSG2 A517V in comparison to DSG2 WT.

Figure S4: Functional studies on DSG2 A517V in comparison to DSG2 WT.

A – Expression of DSG2 WT and A517V as GFP fusion proteins in COS-1 cells; cells transfected with GFP alone served as a control (third lane). The recombinant proteins were detected using a GFP antibody (top) or a DSG2 antibody (bottom, also recognises endogenous DSG2). DSG2 A517V is expressed normally and does not influence expression of endogenous DSG2. Position of marker proteins are indicated, asterisk mark degradation products of DSG2 GFP proteins; note much higher expression of GFP alone. No differences between DSG2 WT and A517V were observed.

B – DSG2 A517V shows normal binding to DSC2: COS-1 cells transfected with DSG2 WT, DSG2 A517V or GFP alone as control (ctr). GFP fusion proteins were immunoprecipitated with a GFP antibody and detected with a DSG2 antibody (top). Immuno-complexes were probe for bound DSC2 (bottom). Binding of DSG2 WT and A517V to DSC2 was comparable.

C – Normal localisation of DSG2 A517V in the cardiac cell line HL-1: DSG2 WT and A517V GFP fusion proteins were transiently expressed in HL-1 cells (top), counterstained with the cell-cell contact marker plakoglobin (middle); merged images (bottom): DSG2 GFP green, plakoglobin red; scale bar represents 10 μm. Both WT and A517V DSG2 were found at the cell-cell contacts and in the Golgi apparatus.

D – Normal localisation of DSG2 A517V in neonatal rat cardiomyocytes: Cells were transiently transfected with DSG2 WT or A517V GFP (first row) and counter-stained with the cell-cell contact marker plakoglobin (second row) and the sarcomeric marker alpha-actinin (third row). Merged images (fourth row: DSG2 GFP green, plakoglobin red, alpha-acitnin blue); scale bar represents 10 μm. No difference in localisation was observed between DSG2 WT and A517V).

None of the experiments identified a pathogenic potential of the DSG2 A517V protein. In agreement with our functional studies, the conservative change DSG2 A517V was predicted to be benign by bio-informatics tools (SIFT 'tolerated' and PolyPhen score 0.426 'benign').

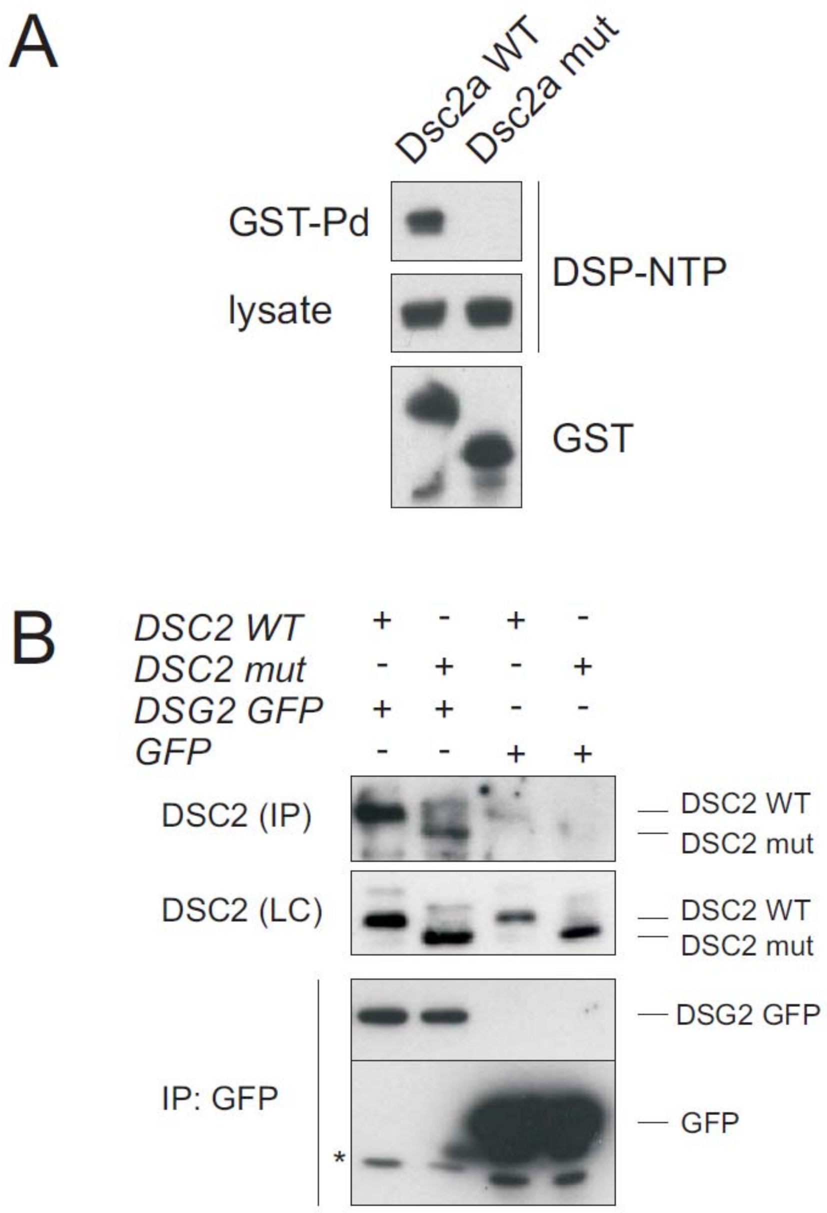

Figure S5: Binding of DSC2a WT and Q851fsX855 mutant to DSP and DSG2.

A – DSC2a WT, but not DSC2a mutant protein bound to an N-terminal DSP construct (DSP-NTP) in GST-pulldown assays. Note that DSC2b does not bind this DSP construct either[6].

B – COS-1 cells were transfected with DSC2 and DSG2 constructs in the indicated combinations (GFP alone serving as negative controls), and GFP fusion proteins precipitated with a GFP antibody. Bound DSC2 proteins were detected by Western blotting (top row), for comparison lysate controls are shown (LC, second row). Asterisk indicates degradation products of DSG2 GFP.

Supplementary Table

Table S1: Clinical evaluation of the index patient and her living daughter

Summary of clinical evaluation and genetic screening of the index patient and her living daughter (see Figure S1), based on the revised Task Force Criteria[8]. Diagnostic and genetic features suggestive for ARVC are printed in bold. (M – major, m – minor criteria). The summary of the number of major and minor diagnostic criteria is given, suggesting a borderline diagnosis of ARVC in both individuals.

	index patient	*daughter*
Age	52	18
Symptoms	palpitations	none
Depolarisation	signal averaged ECG normal<br>**QRS duration 84 msec, >55msec terminal S wave (m)**	signal averaged ECG normal<br>normal QRS duration
Repolarisation	no T-wave inversion	**T-wave inversion V1, V2 (m)**
Functional/Structural (echocardiogram)	normal RV size/function<br>no RV wall motion abnormalities	normal RV size/function<br>no RV wall motion abnormalities
Arrhythmias (24 h ECG monitoring)	no ventricular ectopy or ventricular arrhythmias	no ventricular ectopy or ventricular arrhythmias
Tissue Characterization	more than 75 % residual myocytes	not done
Family History/Genetics	**DSC2 Q851fsX855 (M)**	**DSC2 Q851fsX855 (M)**
Task Force Criteria	**1 M + 1 m**	**1 M + 1 m**
Diagnosis of ARVC	borderline	borderline

Supplementary References

1 Sen-Chowdhry S, Syrris P, McKenna WJ: Role of genetic analysis in the management of patients with arrhythmogenic right ventricular dysplasia/cardiomyopathy. J Am Coll Cardiol 2007; 50:1813-21.

2 Saffitz JE, Green KG, Kraft WJ, Schechtman KB, Yamada KA: Effects of diminished expression of connexin43 on gap junction number and size in ventricular myocardium. Am J Physiol Heart Circ Physiol 2000; 278:H1662-H1670.

3 Gehmlich K, Pinotsis N, Hayess K et al.: Paxillin and ponsin interact in nascent costameres of muscle cells. J Mol Biol 2007; 369:665-82.

4 Geier C, Gehmlich K, Ehler E et al.: Beyond the sarcomere: CSRP3 mutations cause hypertrophic cardiomyopathy. Hum Mol Genet 2008; 17:2753-65.

5 Gehmlich K, Asimaki A, Cahill T et al.: Novel missense mutations in exon 15 of desmoglein-2: Role of the intracellular cadherin segment in arrhythmogenic right ventricular cardiomyopathy? Heart Rhythm 2010; 7:1446-53.

6 Gehmlich K, Syrris P, Peskett E et al.: Mechanistic insights into arrhythmogenic right ventricular cardiomyopathy caused by desmocollin-2 mutations. Cardiovasc Res 2010; doi: 10.1093/cvr/cvq353.

7 Grunnet M, Behr ER, Calloe K et al.: Functional assessment of compound mutations in the KCNQ1 and KCNH2 genes associated with long QT syndrome. Heart Rhythm 2005; 2:1238-49.

8 Marcus FI, McKenna WJ, Sherrill D et al.: Diagnosis of arrhythmogenic right ventricular cardiomyopathy/dysplasia: proposed modification of the task force criteria. Circulation 2010; 121:1533-41.
